# Fungemia With Wickerhamomyces anomalus: A Case Report and Literature Review

**DOI:** 10.7759/cureus.53550

**Published:** 2024-02-04

**Authors:** Yui Sakai, Toshibumi Taniguchi, Yoriko Herai, Misuzu Yahaba, Akira Watanabe, Katsuhiko Kamei, Hidetoshi Igari

**Affiliations:** 1 Infectious Diseases, Chiba University School of Medicine, Chiba, JPN; 2 Infectious Diseases, Chiba University Hospital, Chiba, JPN; 3 Respiratory Medicine, Misato Central General Hospital, Misato, JPN; 4 Clinical Research, Chiba University Medical Mycology Research Centre, Chiba, JPN

**Keywords:** pichia anomala, hansenula anomala, wickerhamomyces anomalus, candida pelliculosa, catheter-related blood stream infection, fungemia

## Abstract

We report the case of an 84-year-old man with a history of IgG4-related sclerosing cholangitis who was diagnosed with advanced esophageal cancer and underwent radiation and chemotherapy. An implantable central venous access port was placed for chemotherapy and total parenteral nutrition. The patient presented with a fever and received antimicrobial therapy for acute cholangitis but remained febrile, and subsequently, yeast was detected in the aerobic bottle of blood culture obtained from the central venous line. The yeast was identified as *Wickerhamomyces anomalus*. Liposomal amphotericin B was administered, and the central line access port was removed. After confirmation of negative blood cultures and 14 days post treatment, he underwent reinsertion of the central line access port. Due to persistent pain at the insertion site, fluconazole was added for an additional 14 days, and the patient was discharged and transferred to another hospital. *Wickerhamomyces anomalus* is a rare fungal infection with other synonyms including *Pichia anomala*, *Hansenula anomala*, and *Candida pelliculosa*. A literature review of 53 case reports of *Wickerhamomyces* *anomalus*, *Pichia anomala*, *Hansenula anomala*, and *Candida pelliculosa* was conducted, with a total of 211 cases reviewed. Fungemia was reported in 94% of cases, with central venous catheterization, parental feeding, low birth weight, and immunocompromised status identified as major risk factors. The majority of cases were pediatric, particularly neonatal, and there were reports of nosocomial infections causing outbreaks, with some cases involving the eye such as endophthalmitis or keratitis.

## Introduction

*Wickerhamomyces anomalus*, also classified as *Hansenula anomala*, is a yeast described initially by Hansen in 1891. Interestingly, this pathogen has other synonyms, including *Candida pelliculosa* and *Pichia anomala*. It colonizes the throat and gastrointestinal tract of humans, although this microorganism has also been isolated from the soil, pigeon droppings, plants, and fruits [1-3]. It is an emerging pathogen found mainly in immunocompromised patients, and little is known about its pathogenicity [4].

*Pichia anomala* has been reported to cause infections, such as endocarditis, urinary tract infections, and fungemia, in immunocompromised hosts, including those with hematologic malignancies and advanced human immunodeficiency virus (HIV). It has also been significantly associated with the use of broad-spectrum antibiotics [3-9].

Here, we report a case in which *Wickerhamomyces anomalus* was identified in a patient with esophageal cancer who was receiving total parenteral nutrition, due to a bloodstream infection from the implantable central venous access port.

## Case presentation

**Figure 1:**
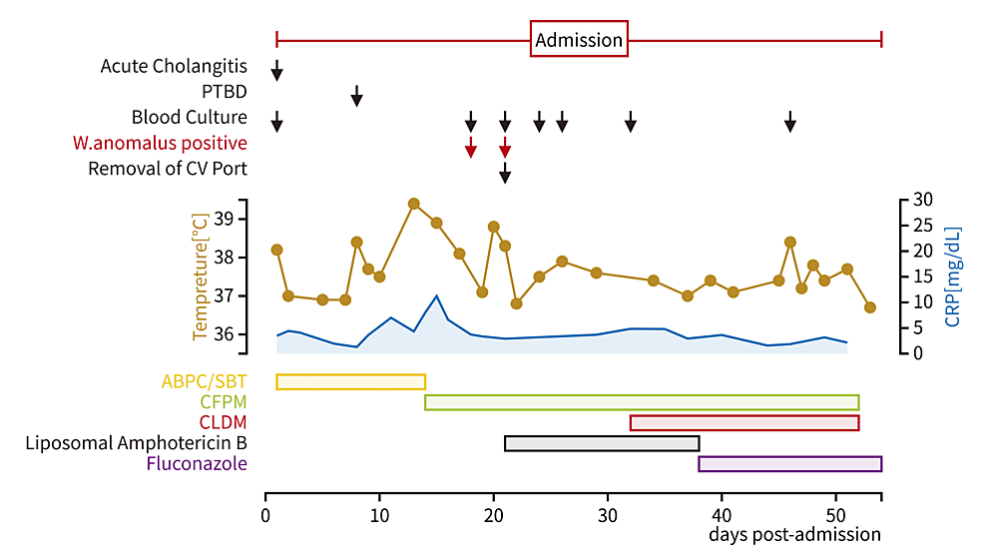
Clinical course post admission PTBD: percutaneous transhepatic biliary drainage; CV: central venous; ABPC/SBT: ampicillin/sulbactam; CFPM: cefepime; CLDM: clindamycin; CRP: C-reactive protein

We present an 84-year-old male with a medical history of IgG4-related sclerosing cholangitis and a bile duct stent. He was diagnosed with advanced esophageal cancer during a stent exchange in April, which occurred six months prior to his current admission. Following the diagnosis, he began radiation and chemotherapy therapy in June, four months before his admission. In August, an implantable central venous access port was placed for the administration of chemotherapy and total parenteral nutrition, two months before his admission. During the course of his treatment, he developed acute cholangitis, characterized by fever, leukocytosis, and abnormal liver function test results compared to his usual values (as shown in Figure 1), which led to his admission.

**Figure 2:**
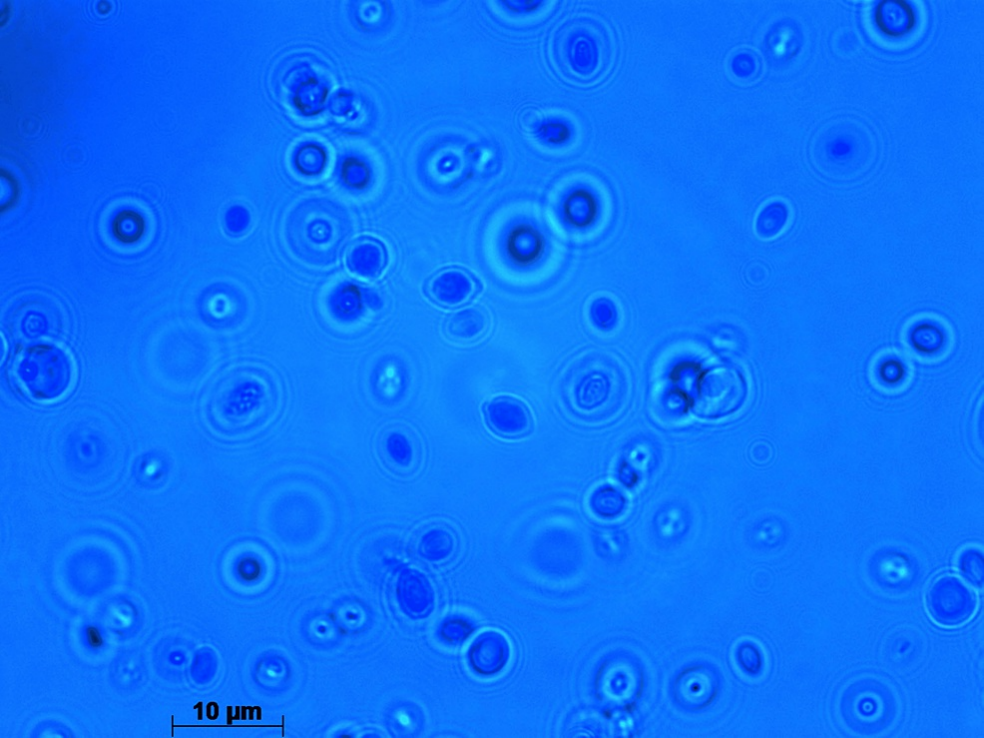
LPCB staining of Wickerhamomyces anomalus LPCB: lactophenol cotton blue

**Figure 3:**
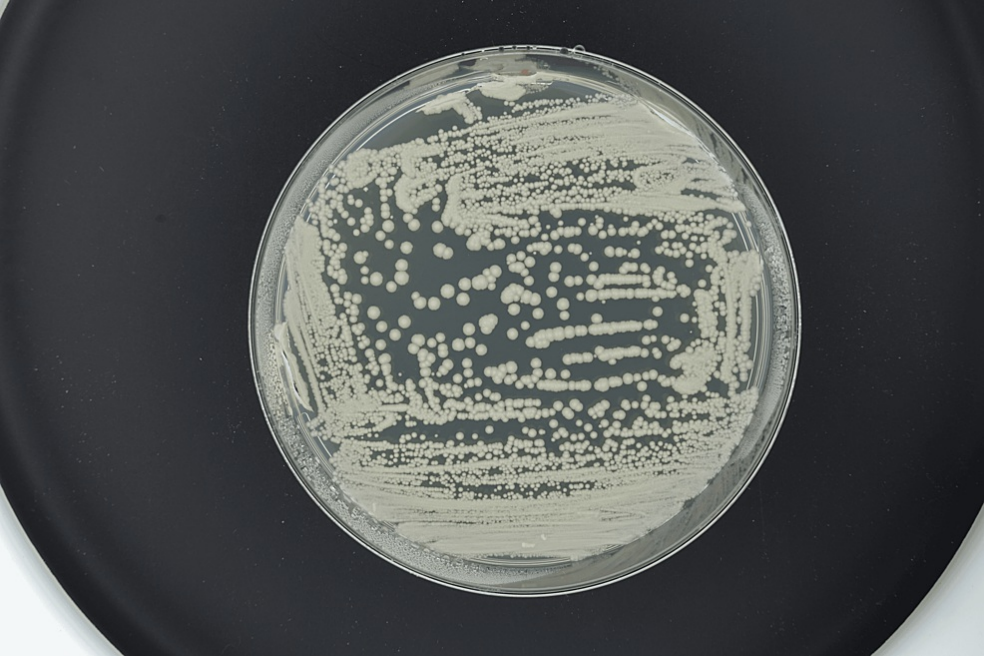
Wickerhamomyces anomalus on potato dextrose agar

**Figure 4:**
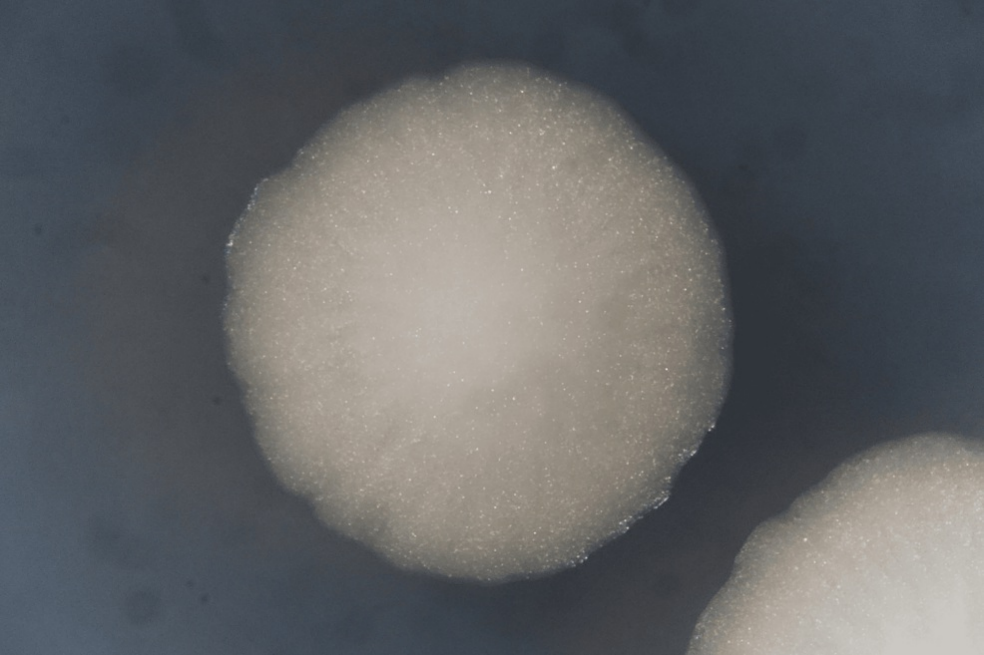
Wickerhamomyces anomalus on potato dextrose agar (enlarged view)

Ampicillin/sulbactam was empirically started which accomplished defervescence. On day 8 of his hospitalization, percutaneous transhepatic biliary drainage (PTBD) was performed. After PTBD, he became febrile again accompanied by increasing abdominal pain. Retrograde cholangitis from PTBD was suspected, and therefore, antimicrobial therapy was switched from ampicillin/sulbactam to cefepime on day 14. His abdominal pain got better; however, he continued to be febrile. On day 18, blood culture was obtained due to suspected refractory cholangitis not responding to antimicrobials. Twenty-one hours later, yeast (Figure 2, Figure 3, and Figure 4) was detected from the aerobic bottle of the blood culture obtained from the central venous access port, which became positive 11 hours earlier than the culture obtained from the peripheral vein.

Analysis with matrix-assisted laser desorption/ionization-time of flight (MALDI-TOF) mass spectrometry revealed that the yeast was *Wickerhamomyces anomalus*. The minimum inhibitory concentration (MIC) indicated that most of the antifungals might be effective (Table 1), but we opted for treatment with amphotericin B due to case reports of successful outcomes. Additionally, at this point, we were uncertain about the involvement of other organs, such as the eyes and urinary tract; therefore, we chose amphotericin B.

**Table 1:** Susceptibility of Wickerhamomyces anomalus MIC: minimum inhibitory concentration; AMPH-B: amphotericin B; 5-FC: flucytosine; FLCZ: fluconazole; ITCZ: itraconazole; MCFG: micafungin; VRCZ: voriconazole; CPFG: caspofungin

Antifungals	MIC (mcg/mL)
AMPH-B	= 0.5
5-FC	≤ 0.125
FLCZ	= 2
ITCZ	= 0.25
MCFG	= 0.03
VRCZ	= 0.06
CPFG	= 0.25

Liposomal amphotericin B was started at 3 mg/kg. On the next day (day 20), he underwent emergent removal of the implantable central venous access port, and the port itself was also sent for culture. The repeat blood culture, as well as the culture from the port, turned out to be positive for *Wickerhamomyces anomalus* as well. Since the nature of this pathogen was similar to that of *Candida* spp., we sought ophthalmologic consultation to rule out endophthalmitis, which was not found. Blood culture was obtained every 48 hours, and the one obtained on day 23 confirmed a negative result. Liposomal amphotericin B was administered for 17 days, 14 days after the confirmation of negative blood culture. His fever and inflammatory markers improved. However, he complained of continuing pain at the central venous access port site, and upon discussion with the primary team, we decided to extend his antifungal treatment with fluconazole for 14 days and discharge him thereafter.

## Discussion

This was a case of *Wickerhamomyces anomalus* catheter-related bloodstream infection in a patient undergoing radiation and chemotherapy for his esophageal cancer. He was successfully treated with liposomal amphotericin B, which achieved prompt negative blood culture.

We reviewed 53 case reports that were available on PubMed for the past 40 years (Table 2): nine reports under *Wickerhamomyces anomalus*, eight reports under *Pichia anomala*, 23 reports under *Hansenula anomala*, and 13 reports under *Candida pelliculosa*. 

**Table 2:** A literature review of Wickerhamomyces anomalus cases M: male; F: female; avg: average; med: median; CV: central venous; IV: intravenous; ICU: intensive care unit; AMPH-B: amphotericin B; L-AMB: liposomal amphotericin B; ABLC: amphotericin B lipid complex; 5-FC: 5-fluorocytosine; N/A: not available; TPN: total parenteral nutrition; MV: mitral valve; CAPD: continuous ambulatory peritoneal dialysis; NICU: neonatal intensive care unit; PICC: peripherally inserted central catheter; N-CPAP: nasal continuous positive airway pressure; HIV: human immunodeficiency virus; AIDS: acquired immunodeficiency syndrome

Number of cases	Age, sex, cases	Geography	Site of infection	Underlying conditions	Treatment	Outcome	Author, year, reference no.
Case reports under *Wickerhamomyces anomalus*							
1	5, M	Iran	Fungemia	Griscelli syndrome with hemophagocytic syndrome	AMPH-B	Died	Aboutalebian et al., 2023, [10]
1	N/A	Spain	Postoperative endophthalmitis	Cataract surgery	Voriconazole	Cured	Galván Ledesma et al., 2023, [11]
13 (outbreak)	29-89, avg 57, M6, F7	China	Fungemia	CV placement, broad-spectrum antibiotics, TPN, MV	L-AMB	Died 5, cured 8	Zhang et al., 2021, [12]
2	0, M1, F1	Indian	Fungemia	Low birth weight	AMPH-B	Cured	Shubham et al., 2021, [13]
1	48, M	Turkey	Peritonitis	CAPD	Fluconazole and anidulafungin	Cured	Evren et al., 2021, [14]
1	36, M	India	Fungemia	Leukemia, long-term CV catheter	Fluconazole	Cured	Mehta et al., 2020, [15]
1	0, F	Brazil	Fungemia	CV placement, enteral nutrition, pericardiocentesis	AMPH-B	Died	Dutra et al., 2020, [16]
1	0, M	Turkey	Fungemia	Pneumonia and sepsis	Fluconazole	Cured	Yilmaz-Semerci et al., 2017, [17]
1	91, F	Japan	Keratitis	Corneal transplantation	Fluconazole	Cured	Kamoshita et al., 2015, [18]
Case reports under *Pichia anomala*							
1	21, M	USA	Fungemia	Sickle cell disease	Fluconazole	Cured	Chan et al., 2013, [19]
1	12, F	Japan	Pneumonia	Acute lymphatic leukemia	Micafungin, AMPH-B	Cured	Sugano et al., 2010, [20]
1	50, F	Korea	Keratitis	Systemic lupus erythematosus and Stevens-Johnson syndrome	AMPH-B	Cured	Park et al., 2008, [21]
2	Adult, F2	Brazil	Fungemia	Nosocomial infection	AMPH-B, 5-FC	Died 2	Paula et al., 2006, [22]
17 (outbreak)	Med. age 1.1, M9, F8	Brazil	Fungemia	CV placement	AMPH-B, fluconazole	Died 7, cured 10	Pasqualotto et al., 2005, [23]
5	0, 0, 0, 8, 14, M4, F1	Turkey	Fungemia	CV placement	AMPH-B	Died 1, cured 4	Bakir et al., 2004, [24]
379 (outbreak)	Children	India	39.8% fungemia	First found in the neonatal ward, spread to other pediatric wards	Fluconazole, AMPH-B	Died 42.4%	Chakrabarti et al., 2001, [25]
4 (outbreak)	0, M3, F1	Brazil	Fungemia	ICU	AMPH-B, 5-FC	Cured	Aragão et al., 2001, [26]
Case reports under *Hansenula anomala*							
1	70, M	Korea	Arthritis	Diabetes mellitus	AMPH-B	Cured	Choi et al., 2010, [27]
5	4 adults, 1 newborn	Argentina	Fungemia	Corticoids, catheters, surgical procedures, and neutropenia	N/A	Died 2, cured 3	Mestroni and Bava, 2003, [28]
1	32, M	USA	Pneumonia, fungemia	Motor vehicle accident	ABLC	Cured	Kane et al., 2002, [29]
8 (outbreak)	Adults, M5, F3	Croatia	Fungemia	Surgical ICU	Fluconazole, miconazole	Died 3, cured 5	Kalenic et al., 2001, [30]
1	0, F	Malaysia	Fungemia	Low birth weight, parental nutrition	AMPH-B	Died	Wong et al., 2000, [31]
1	0, N/A	Taiwan	Fungemia	Premature	Fluconazole, AMPH-B	Cured	Ma et al., 2000, [32]
24 (outbreak)	Med. age 11, M11, F13	Brazil	Fungemia	Oncology and transplant unit	AMPH-B	Cured	Thuler et al., 1997, [8]
1	61, M	Japan	Fungemia	Cancer and parental nutrition	Fluconazole	Cured	Sumitomo et al., 1996, [6]
1	46, M	Slovakia	Fungemia	Leukemia, CV placement	AMPH-B	Cured	Kunová et al., 1996, [33]
4	0, 0, 4, 8, M2, F2	Japan	Fungemia	Malignancy, CV placement	Fluconazole	Died 1, cured 3	Yamada et al., 1995, [3]
1	22, M	Australia	Fungemia	Bone marrow transplant, CV placement	AMPH-B	Cured	Goss et al., 1994, [34]
1	4, M	USA	Fungemia	Motor vehicle accident, CV placement	AMPH-B	Cured	Alter and Farley, 1994, [35]
1	0, F	Canada	Fungemia	Premature, CV placement	AMPH-B	Cured	Sekhon et al., 1992, [36]
1	63, M	Japan	Fungemia	Lung cancer, CV placement	Fluconazole	Cured	Hirasaki et al., 1992, [7]
3	0, 0, 15, M1, F1, N/A 1	Israel	Fungemia, peritonitis	Dialysis, CV placement	AMPH-B	Cured	Moses et al., 1991, [9]
2	48, M	Spain	Fungemia	Leukemia, CV placement	AMPH-B	Cured	López et al., 1990, [5]
1	51, F	Spain	Fungemia	Leukemia, CV placement	AMPH-B	Cured	Muñoz et al., 1989, [37]
1	10, M	USA	Fungemia	Low birth weight, parental nutrition	AMPH-B	Cured	Dickensheets, 1989, [38]
1	32, M	Saudi Arabia	Urinary tract infection	Renal transplant	None	Cured	Qadri et al., 1988, [39]
2	55, 59, F2	USA	Fungemia	Endometrial cancer, ovarian cancer	AMPH-B	Cured	Klein et al., 1988, [40]
1	34, M	USA	Fungemia	Hematologic malignancy, CV placement	AMPH-B	Cured	Haron et al., 1988, [41]
1	40, M	USA	Fungemia, endocarditis	Bicuspid valve, IV drug use	AMPH-B	Cured	Nohinek et al., 1987, [42]
8 (outbreak)	0, N/A	UK	Fungemia 7, meningitis 1	NICU	AMPH-B, 5-FC (one with no treatment)	Died 2, cured 6	Murphy et al., 1986, [2]
Case reports under *Candida pelliculosa*							
14 (outbreak)	0, M8, F6	China	Fungemia	Low birth weight, catheter-related	Fluconazole	Cured	Yang et al., 2021, [43]
1	0, M	China	Fungemia	Low birth weight	Fluconazole	Cured	Cai et al., 2021, [44]
1	75, F	Korea	Arthritis	None	Micafungin, fluconazole	Cured	Song et al., 2020, [45]
11 (outbreak)	Adults, M5, F6	Korea	Fungemia	ICU	Micafungin, caspofungin	Died 4, cured 7	Jung et al., 2018, [46]
1	57, M	Turkey	Endophthalmitis	Cataract surgery	AMPH-B	Cured	Esgin et al., 2014, [47]
6 (outbreak)	0, M3, F3	Taiwan	Fungemia	PICC 3, N-CPAP 3	AMPH-B, fluconazole	Died 1, cured 5	Lin et al., 2013, [48]
5 (outbreak)	0, M2, F3	Brazil	Fungemia	Parental nutrition	Fluconazole, AMPH-B	Cured	da Silva et al., 2013, [49]
1	46, M	UK	Meningitis	HIV	Unknown	Died	Ratcliffe et al., 2011, [50]
4 (outbreak)	0, unknown	Turkey	Fungemia	CV placement, pediatric ICU	Fluconazole, AMPH-B	Cured	Kalkanci et al., 2010, [51]
3	0, 0, 8, sex N/A	Slovakia	Fungemia	Cardiac surgery	AMPH-B	Cured	Krcmery et al., 2009, [52]
1	0, F	Slovakia	Fungemia	Hemodialysis, CV placement	Fluconazole	Cured	Hanzen and Krcmery, 2002, [53]
1	N/A	Germany	Fungemia	Acute pancreatitis	N/A	N/A	Neumeister et al., 1992, [54]
1	N/A	Germany	Fungemia	HIV/AIDS	N/A	N/A	Salesa et al., 1991, [55]

These 53 articles included a total of 211 cases. Among the 211 cases, 154 cases (73%) were pediatrics, mostly including neonates. Twelve articles reported on nosocomial infections causing outbreaks [2,8,12,23,25,26,30,43,46,48,49,51]. Fungemia was reported in 199 cases (94%), and four cases were eye involvement such as endophthalmitis [11,47] or keratitis [18,21]. Major risk factors for fungemia include central venous catheter insertion with parenteral nutrition, low birth weight, and being immunocompromised, such as having a hematologic malignancy [3,5,15,20,33,34,37,41]. Additionally, the use of broad-spectrum antibiotics may also be a risk factor for *Wickerhamomyces anomalus* fungemia [16].

Most patients were treated with either amphotericin B or fluconazole, which seemed to result in the clearance of fungemia and cure. In terms of susceptibility to antifungals, since there are no defined cutoffs in the Clinical and Laboratory Standards Institut (CLSI) and European Committee on Antimicrobial Susceptibility Testing (EUCAST) guidelines, we adopted the susceptibility profile from da Matta et al. [56], specifying amphotericin B ≤ 1 mcg/mL, fluconazole ≤ 8 mcg/mL, itraconazole ≤ 0.12 mcg/mL, voriconazole ≤ 1 mcg/mL, and caspofungin ≤ 1 mcg/mL. Among the extensively used antifungals, which include amphotericin B and fluconazole, caution is advised in the selection of antifungals since some isolates appear to have a higher MIC to fluconazole, potentially leading to treatment failure. In this context, amphotericin B generally appears susceptible and may be preferable when susceptibility is unknown. However, even with amphotericin B, clinical failures have been documented [31], so one should closely monitor its response to treatment.

It is important to highlight an incident in which a pathogen triggered an outbreak of 379 fungemia cases within the pediatric ward of a hospital [25]. Given the vulnerability of the affected individuals, a case-control study of this incident revealed a case fatality rate of 42%, which is alarming. Rapid infection control measures should be implemented, and early treatment should be initiated. If appropriate measures are not taken, this case report of an outbreak demonstrates that disastrous results can occur.

In our case, *Wickerhamomyces anomalus* fungemia occurred in an elderly patient with cancer, who was receiving total parenteral nutrition through an implantable central venous port. The risk factors included being an immunocompromised host, the use of a central venous line, and, finally, parenteral nutrition, which increased the risk for this pathogen. These factors are similar to the risks associated with candidemia. The MIC for amphotericin B was 0.5 mcg/mL, indicating excellent susceptibility. Similarly, susceptibility to fluconazole was measured at 2 mcg/mL, suggesting it would be effective if used initially. Based on the results of the susceptibility testing, we confidently switched to oral fluconazole for extended treatment. Echinocandins also showed high susceptibility. However, due to the poor distribution of echinocandins to the eyes and urinary tract, and with the potential for eye and urinary involvement which was unknown at the time of detection, we opted for either amphotericin B or fluconazole. 

Our investigation mainly focused on this specific mycotic species, *Wickerhamomyces anomalus*, due to its relative obscurity and infrequency in clinical encounters. This necessitates a deeper understanding and refined management strategies among healthcare providers. Although this fungus is rare, its repeated occurrence in past literature emphasizes the importance of this organism. By offering a detailed summary of this lesser-known fungal infection, this paper aims to provide direction for future clinical practice. One should be aware of this pathogen in low birth weight neonates and immunosuppressed patients, especially those receiving total parenteral nutrition through a central venous catheter. Broad-spectrum antibiotics also appear to predispose to* Wickerhamomyces anomalus* infection. When blood cultures indicate the presence of *Wickerhamomyces anomalus*, initiating treatment with amphotericin B or fluconazole is recommended. As *Wickerhamomyces anomalus* is similar to *Candida* spp., we should adopt the same treatment strategy until further differences are elucidated. Although *Wickerhamomyces anomalus* is uncommon, its likelihood increases in healthcare environments housing a considerable number of immunocompromised individuals, thereby potentially leading to outbreaks. Hence, it is of paramount importance for healthcare providers to maintain vigilance and implement stringent hygiene practices.

## Conclusions

This study focuses on *Wickerhamomyces anomalus*, a rare fungus that causes infections mainly in pediatric and immunocompromised patients. Central venous catheters and immunocompromised conditions such as leukemia could be risk factors for this infection. Effective treatments include amphotericin B and fluconazole. The findings highlight the need for increased awareness and strict hygiene in healthcare settings to prevent outbreaks, especially among vulnerable patient groups.
